# Periscope

**Published:** 1881-05

**Authors:** 


### PERISCOPE.

*North American Journal of Homœopathy* February.

Dr. Lawton writes upon Therapeutic Force and explains why in the trituration of a drug, the power of the drug is developed and not also that of the vehicle—say sugar of milk. When a substance is triturated by itself there is a limit to its divisibility When triturated in the presence of another substance as vehicle, its particles are separated and by the vehicle are *kept* separated until the point is reached at which medicinal power is developed. This is a complete answer to Dr. Dake.

Dr Hale reports two cures of Hemicrania with nickel-sulph.

"S. L." gives an excellent clinical review of Argentum nitricum.

Dr. Price reprints a society report upon "Medical Progress" n which he gives a scathing review of the inconsistencies of the old school. At the close of his article he advises the beginner in homœopathy to read the writings of Neidhard, Dunsford, Hughes, &c., expounding the law of cure and *then* when these works are "thoroughly read" to take Hahnemann's Organon!! Astonishing advice! *Hahnemann* first gave to the world the law of cure. *He* first pointed out the way of escape from the confusion that reigned in medical knowledge. *He* first indicated the possibility of *certainty* in the treatment of the sick. These principles he explains in his book "The Organon." After him came a swarm of followers who understood the principles he laid down more or less—generally less. They hastened to put upon paper what they understood or didn't understand concerning this medical philosophy. And these weakling imitations, these mere commentaries upon the original, are recommended to be read before that great original itself!

The original is the announcement of the discovery of a great natural law of cure arrived at by careful experiment—just as other natural laws are discovered by experiment in the physical laboratory.

Pharmacodynamics is the record of an individual *opinion* as to the value of this discovery; This opinion being warped, twisted and distorted by the exigencies of the struggle for existence of its author.

The Organon is a record of pure inductive science.

Pharmacodynamics is an encyclopedia of plausible arts and expedients by which, in the opinion of its writer, a man may best "get on" in the world.

The Organon was written by the individual best qualified to do so—the great discoverer himself.

Pharmacodynamics was written by a professed "follower" of the master.

Yet it is recommended that this book of this *soi-disant* follower of Hahnemann shall be read *before* the work of Hahnemann himself!

If a man earnestly wish to understand homœopathy let him read the Organon. If he be indifferent to the truth let him read Pharmacodynamics.

Dr. Buscher's report of cases of poisoning by Aconitium Nitricum Gallicum is translated from the German for this No.

Dr. Bernard's recommendations of Lachnantes for Phthisis Pulmonum are translated from *Revue Hom. Belge.*

"S. L." gives a translation of a case treated by Dr. Seither. The patient had severe Gastralgia. This was relieved immediately by Nux V. 30. The indications were: *very sensitive, irritable, and easily angered, cannot bear strong light or loud talking. From any mental emotion or a full meal burning and pressure in pit of stomach.* Another attack of the same trouble was finally cured by Carbo-veg. 30. Indications, *abdomen inflated and hard as a drum, with difficult breathing burning and pressing in stomach, relieved by copious stool and discharge of much noisy flatus.*

In commenting upon the above, "S. L." gives a case of aneurism of the right iliac artery cured by Lyc. 30. The indications leading to the use of Lyc. in that particular case are not given.

*American Homœopath*, February.

Dr. Ricardo cured a boil on the face with Arn. 30. No special indications. A case of severe pain in left side of face with redness and swelling of left side of nose and *very irritable*, cured with Cham. 30.

Dr. Meurer treated with success a case of biliary calculi. When called in, after repeated failures of physicians of the old school, ordered a reform in the diet before undertaking drug treatment, the patient meanwhile taking powders of sac. lac. By this most excellent precaution "her case crystalized from out a chaos of symptoms, into a clear form." He then gave Podophyl. Unfortunately he has given no indications. When attack of colic came on, he gave Nux V. 10th decimal with application of hot wet cloths. She soon began to improve and in a few months passed gall stones, and her general health became good.

Dr. Boocock writes upon diphtheria and advises alteration of remedies. He claims success with 150 cases. How can this be, especially as he alternates?

Dr. Ricardo gives a case of incipient phthisis cured by Phos., Sulph., Bry., *and* Scott's emulsion of hypophosphites! The doctor thinks he knows just how far the hypophosphite was useful. How can he be certain of this unless he had studied provings of "Scott's emulsion"? How could he know what remedy cured so long as he gave the emulsion at the same time as the *supposed* similar remedy! How does he know that the improvement at any given time was not owing to this emulsion *instead* of the assumed simillimum? Hahnemann offered a method of prescribing by which such questions as these would be excluded, and the possibility of tracing the improvement to its right source could be increased, and by which the prescriber would *learn* something that would enable him to *repeat* the successful result upon another patient. Let it be understood that the writer is not denouncing the use of "Scott's emulsion", but is simply protesting against using it without having at hand its recorded proving and against giving it *at the same time* that some other remedy is prescribed.

Dr. Morgan shows that grapes cause suppression of urine. He therefore gives them to a patient suffering from profuse urination! ! Is this a case of cure from homœopathicity of the remedy, or is it a case of temporary suppression from an antipathic action?

Dr. Morgan gives also the following indications:

Pleurodynia; Merc.-viv; for *soreness on percussion or pressure of the rib bones or periosteum of the ribs.*

Arn. or Puls. for soreness of the intercostal muscles (not the bones).

Ratanhia, infantile diarrhœa, painless, watery and fetid.

Ferrum-phos., hepatic or gastric pains occurring about bed-time.

Magnes-phos., colic of new-born infants.

Magnes-phos., infantile convulsions. After the spasm excessive sensitiveness to every impression on the senses, even to touch, and especially to noise; look of suspicion and fear; easily agitated.

Acon. infantile diarrhœa; vomiting and purging, the latter always like a "blast of wind and water."

Dr. Lee reports two cases of venereal disease cured respectively by Apis and Nitric Acid. Unfortunately the indications are not very clearly stated.

Dr. Williams reports the cure of a case of cholera with Zincum sulphuricum.

Dr. Hale cured a case of subacute myelitis with Strychnia in doses of about one quarter of a grain.

Dr. Detwiler cured a case of purpura hemorrhagica with

Terebinthina. This drug is stated to have caused hæmaturia, burning in epigastrium and livid spots on the skin of the back and abdomen. These were the indications for its use in the above case.

Dr. Elliott gives an article upon malignant pustule with cases cured by administering Silicea and Lachesis.

"C. L. J." cured a case of typhoid fever with Lyc. on the indication *aggravation of fever between* 5 *and* 6 *p. m.*

Dr. Brigham cured a case of indigestion with constipation and impotence with Phosphorus following Colocynth. Principal symptoms were: griping pains in stomach better from bending forward. Vertigo especially from looking over the left shoulder. Stiffness of nape of neck; stool—which was narrow and scanty. Loss of flesh. Irritability.

The same authority relates a case of vertigo so violent as to cause falling and, momentary unconsciousness. Gets cramps and attacks of catalepsy. During vertigo walks as if intoxicated, has attacks of deafness with blindness. Flatus distends the stomach almost to bursting and goes off explosively. Better in the open air. Lachesis 2c, cured.

Dr. Ockford writes a *homœopathic* paper on Neurasthenia in which he strongly insists upon individualization, the simillimum, and attention to mental symptoms.

From the *Hom. World* is translated a clinical case by Dr. Bernard—Hardenpoint of prurigo of three years standing which had resisted all treatment. The patient was given Rumex Crispus on the indication *itching worse from cold, better from warmth, especially of the bed.* Cured.

Dr. Burnett cured a case of diurnal incontinence of urine with Selenium on indication *involuntary dribbling of urine while walking.* The urine was *red.*

Dr. Ivatts relates a remarkable case of lupus of the face which was completely removed by Calotropis Gigantea. The disease was transferred to the foot where a great discharge took place. Later an old lesion of the lung broke out, the man spat up blood and pus, then fell into the hands of an allopath, then—died.

Dr. Clarke cured a case of enlargement of the spleen with Ceanothus. Principal symptoms were: aching in left side in region of sacro-iliac joint and extending up to the fifth rib. Sensation of dropping water under the arm.

From the *Hom. World* is copicd a case of diarrhœa of seventeen years standing cured with Jalap. Principal symptoms were: six or eight stools in 24 hours. Stools dark, thin like gruel, offensive, attended and followed by griping and tenesmus.

*The Medical Advance*, February.

Dr. Brigham writes upon Gynæcology. He reports the cure of a case of uterine polypus with Thuja 2c. Principal symptoms were: sallow complextion, aggravation from emotion. flushes of heat, feeling as if she would die, temperament lymphatic, sanguine.

From *Die Allg. Hom. Zeitung* is translated an article by Dr. Goullon entitled "What Dose of Sulphur shall be administered." He gives five clinical cases cured with sulphur,—dyspepsia, ascites, aphonia with choking cough, epilepsy, mange in a dog, and albuminuria with dropsy.

The *Advance* has added to its pages a department devoted to the microscope under the editorship of J. Edwards Smith, M. D. This department leads off with an article of Prof. Dolbear "Upon the Absolute Invisibility of Molecules and Atoms.,' He concludes that from the swift molecular motion "it must be forever impossible to see atoms or molecules."

Dr. Kilgour relates a case of caries of the vertebræ cured by giving first Sil. then Hepar, and later the two in alternation. The alternation is the one objectionable thing in this treatment. It is impossible to tell which cured. There is therefore no lesson learned by the reader that *he* may go and do likewise. It is always better to give only one remedy at a time; we are then able to come to a definite conclusion. It should always be borne in mind that in treating the sick as in investigating nature in the physical laboratory, we must reduce the unknown factors to as small a number as possible. More than one half of the appliances of the physicians are with a view of this very thing—excluding all factors but one, and then watching the operation of that one factor in order to weigh its influence in the creation of the phenomenon that may happen to be the subject of research.

Dr. Ryall gives a case of hemorrhage from the lungs with a history showing phthisis, which was much benefitted by Psorinum 200.

*The Homœopathic Times*, March.

Dr. Mitchell gives an article entitle "The Experiment of Allopathic Homœopathy." It is a very instructive lesson and should be read by all homœopathists.

Amidst the "din with which the air is flllled" of "cries of regular and irregular" amidst a shower of parthian arrows, anointed with the "taunts of mongrel and renegrade," and hurled from within the "impregnable fortress" of the "International Societies, True Hahnemannians, and Rolls of Honor." *The Times* dares to "state its convictions as to what constitutes a *true physician.*" Under such circumstances it is not surprising to find its ideas a little mixed. For axample it "denies the right of any man or set of men to tack on to a principle, which has been clearly and distinctly formulated, theories having no connection with it and which by no process of reasoning could be evolved from it." That is pecisely what the Internationals are fighting to maintain. It is the "impregnable fortress" that they are determined to hold. Who are the philosophers who do "tack on" "therories that have no conndction with it"? We will answer. They who, in accordance with the "freedom" claimed, give a dose of morphia for pain because they are too inefficient or too lazy to study the right remedy; and then justify this eclectic practice by an appeal to rationalism, "supplementary principles," etc. Is not the man who does this, guilty of "tacking on" theories that have no connection with it"?

When a man gives fifteen grains of quinine for intermittent fever instead of comparing the totality of the symptoms; and brazenly claims it to be in accordance with the law of the similars, notwithstanding the fact apparent to every one that he has given it empirically, is not such a man guilty of "tacking on theories that have no connection with it"? Who are they who advocate the measures here stated as a part of the duties of the "true physician" and in the same breath denounce the "tacking on" of "theories that have no connection with" the law of similars? They are the editors of *The Times* and certain of its contributors. In view of these facts does it not seem a little odd to find *The Times* denouncing any thing in the way of eclectic practice?

"E. W. E." writes upon "Dynamists." He claims that there are *three* parties in the homœopathic school—materialists, "altiosimists," and dynamists. The first give medicines only in those dilutions that can be proved under the microscope to contain particles of the drug. The second give only high and fluxion potencies, whilst the third occupy a middle ground and give up to the thirtieth potency.

Dr. Taylor writes upon "Hahnemann's Law of Dose," in which he endeavors to prove that Hahnemann advocated the use of medicines in quantity sufficient to act with greater intensity than the original disease.

Inasmuch as the "Internationals" do not deny this, there can be but one motive in bringing forth this argument for their inspection, and that is a desperate effort to find an excuse for giving fifteen grains of quinine in intermittent fever. When a man is too ignorant or too lazy to find the remedy he wants, "freedom" to give a massive dose of quinine or some other empirically-prescribed medicine and then to have the whole thing glossed over by calling the suppression of the chill (in intermittent fever) a cure; arguing that it *must* be homœopathic because it *did* "cure," and then justifying the excessive quantity given by an appeal to "Hahnemann's law of dose." Thus is the conscience of the doctor quieted and he is at the same time relieved from the attacks of the dreaded "Internationals."

*Hahnemannian Monthly*, March.

Dr. Shearer writes upon Cyclamen Europeaum, and gives a clinical case of hemeralopia or night blindness cured by it. The principal indication was *distressing vertigo coming on as soon as it was dark.* Cyclamen 30 cured in about six weeks.

Dr. Dunning writes upon Psoriasis and gives a clinical case cured with Ars. Iod. 4 x in about eight weeks.

Dr. Nichols reports a case of abdominal typhus fever which "several 'leading' physicians succeeded in pushing into narcotism and joined in a fatal prognosis." Diarrhœa painless, clay-colored, thin, copious frequent. *Vomiting everything at once but hungry while nauseated.* Verat. 2 C. cured in two or three days.

Dr. Paris G. Clarke gives three cases. One case, headache if the patient sleeps on his back. *Offensive stools in the morning, hurrying him out of bed.* Sulphur 30 M. cured.

The second case—chronic diarrhœa was cured with Nux v. C.C. followed by Podophyl.

The third was a case of painful and difficult urination which caused cold sweat on the forehead. Cured with Nux v. 2 C. foloped by Sulph. C.M.

Dr. Price writes upon "Fluxion Dilution." He declares that "we are guilty either of ignorance or wilful perversion of truth when we publish cures of cases by fluxion dilutions.'' The old school have always denied every claim made by homœopathists, and attributed our cures to "imagination." Dr. Price repeats the old comedy of the allopathists when he denies the potencies being beyond his power to understand or explain and directly opposed to his prejudices, just as the entire philosophy of homœopathy is to the old school, he, like them attributes the results to imagination.

*Amercan Observer*, February and March.

Drs. Houghton and Morton write upon acute catarral inflammation of the middle ear, and give indications for several remedies. The general editor gives a statement of his "position." He compares the "exclusives" to pharisees! That is to say that if a man proclaims himself a homœopath and then use old school measures he must be allowed to do so and no voice must be raised against it on penalty of being called "Pharasee" There is no law that compels a man to assume the title of homœopath merely because he *occasionally* uses a homœopathic method of practice. Why then do gentlemen of this mode of thought allow this title to be applied to them? The answer is plain! There's money in it! When the editor raises the dose question, he raises a perfectly false issue. Dose has nothing to do with it. A man who gives high potencies is not necessarily a homœopath, and a man who gives crude drugs is not necessarily a "mongrel." What the "exclusives" denounce and will continue to denounce is the empirical exhibition of quinine in massive doses in order to *cover up* (suppress) the manifestation of a malarial chill; the giving of morphine to "allay pain;" of chloral to "procure sleep" and of purgatives to "clear out the bowels."

The calling of this practice homœopathic and the styling of such practitioners homœopathists. If men who believe in eclectic practice will but honestly call themselves such, they are entitled to a hearing. But when they continue to claim fellowship in a system they do not practice and have no sympathy with, they are not in a moral attitude and therefore can not be considered respectable people.

Dr. Henry asserts that he cures cases of trismus of children with nitrite of amyl. He gives no indications, however, so that we are reduced to the supposition that *all* cases must be treated the same way. If so why are not *all* cases cured seeing that the old school are well aware of the use of amyl for this purpose? He quotes a paper of the late Dr.

Hering in support of his views. But Dr. Hering advocated it only in cases that from bad treatment were certain to die. It was then in view of *euthanasia* that Dr. Hering, proposed to use amyl. Dr. Henry, however, takes advantage of Dr. Hering, quoting him, in support of his own unhomœopathic practice.

Dr. Henry proposes the use of horse-raddish for milky urine in children. He gives no indications but quotes the clinical experience of an old woman and appeals to the chemical composition of the *ash* as an argument for its use. Does he not know that this remedy was proved by Dr. Lippe in 1846? Similarly without any previous provings, he proposes aralia for otorrhœa, turpeth mineral for croup, creasote for "throat disease," benzoic acid in "urinary derangements", picric acid for chapped nipples, atropia for failing circulation, epilepsy, &c. Not one of these drugs has any indications given for it except possibly a few *general* symptoms for one of them. The natural inference is that he expects them to cure *every case*. If this be so there is no excuse for the existence of the doctrine called homœopathy. W. M. J.

